# Ecologically-Relevant Maps of Landforms and Physiographic Diversity for Climate Adaptation Planning

**DOI:** 10.1371/journal.pone.0143619

**Published:** 2015-12-07

**Authors:** David M. Theobald, Dylan Harrison-Atlas, William B. Monahan, Christine M. Albano

**Affiliations:** 1 Conservation Science Partners, Truckee, California, United States of America; 2 Department of Fish, Wildlife, and Conservation Biology, Colorado State University, Fort Collins, Colorado, United States of America; 3 Graduate Degree Program in Ecology, Colorado State University, Fort Collins, Colorado, United States of America; 4 Inventory and Monitoring Division, National Park Service, Fort Collins, Colorado, United States of America; 5 John Muir Institute of the Environment, University of California Davis, Davis, California, United States of America; Technion -- Israel Institute of Technology, ISRAEL

## Abstract

Key to understanding the implications of climate and land use change on biodiversity and natural resources is to incorporate the physiographic platform on which changes in ecological systems unfold. Here, we advance a detailed classification and high-resolution map of physiography, built by combining landforms and lithology (soil parent material) at multiple spatial scales. We used only relatively static abiotic variables (i.e., excluded climatic and biotic factors) to prevent confounding current ecological patterns and processes with enduring landscape features, and to make the physiographic classification more interpretable for climate adaptation planning. We generated novel spatial databases for 15 landform and 269 physiographic types across the conterminous United States of America. We examined their potential use by natural resource managers by placing them within a contemporary climate change adaptation framework, and found our physiographic databases could play key roles in four of seven general adaptation strategies. We also calculated correlations with common empirical measures of biodiversity to examine the degree to which the physiographic setting explains various aspects of current biodiversity patterns. Additionally, we evaluated the relationship between landform diversity and measures of climate change to explore how changes may unfold across a geophysical template. We found landform types are particularly sensitive to spatial scale, and so we recommend using high-resolution datasets when possible, as well as generating metrics using multiple neighborhood sizes to both minimize and characterize potential unknown biases. We illustrate how our work can inform current strategies for climate change adaptation. The analytical framework and classification of landforms and parent material are easily extendable to other geographies and may be used to promote climate change adaptation in other settings.

## Introduction

The science underpinning climate adaptation planning is nascent and complex, but recent guidance emphasizes the need to characterize environmental heterogeneity that underlies a variety of strategies to conserve ecological patterns and processes in the face of climate change (e.g., [1,2,3,4]). One of the increasingly used strategies is to characterize environmental heterogeneity by identifying “arenas of biological activity,” “land facets” or physiographic settings (e.g., [5,6,7]). Of the general adaptation strategies identified by climate-smart conservation [3], protecting different components of environmental heterogeneity forms the basis for at least four of them. One key strategy is to *protect key ecosystem features* ([4,8,9,10,11]), because areas with high environmental heterogeneity support high genetic and species diversity ([3,5,12,13,14,15,16), including rare plant species of conservation concern [7]. A second strategy is to *support evolutionary potential* by representing a variety of environmental settings, which facilitates adaptation of populations and reassembly of communities [17], and species’ responses to climate change [18,19]. A third strategy is to *protect refugia*—places that are less likely to be influenced by climate change because site-level climate conditions are decoupled from changes in regional climate [20]. A fourth strategy is to *ensure connectivity* among different habitat types and physiographic settings, and many of the landscape features such as riparian zones that are important for connectivity are strongly aligned with certain landforms (e.g., valley bottoms).

Here we describe the development of a novel classification and maps of landforms and physiography to be used as foundational data for ecological modeling of species and biodiversity to inform climate adaptation planning activities. We recognize that physiographic settings can be considered over a variety of spatial and temporal scales, and that some landforms (especially locally) can change due to extreme events, such as flash floods or earthquakes. This has led scientists to view geomorphology as non-linear and dynamic [21]. Yet, here we generalize these dynamics to emphasize geophysical settings that occur over broad spatial extents (>100 km^2^) and change, on average, at relatively slow time scales (centuries to millennia). In comparison to the dramatic climatic and biotic responses that are forecast to occur due to climate change [22], we view these settings as relatively static. Our characterization of landforms confers a practical advantage for climate adaptation because it isolates those features that will likely remain on the landscape and allow managers to examine and focus on the relatively high uncertainty in dynamic variables, such as climate model projections of temperature or precipitation.

Seminal work by ecologists has long recognized that landforms [23] structure ecological patterns and processes (e.g., [24–27]). More recently, scientists have brought this concept into conservation planning by identifying land facets [6,28], abiotic units [29–32], geodiversity [33,34], geomorphological units [35], and the geophysical stage [7,36]. These physical features have the potential to inform climate adaptation strategies by helping characterize and interpret how species’ distributions are locally influenced by climate and other broad scale dynamic factors. Importantly, not all species are expected to interact with the physiographic setting in the same way. Species’ ecological niches (i.e., the environmental conditions and resources required by species for survival) are shaped in part by biophysical variables (e.g., temperature), which are in turn shaped by physical variables that define the physiographic setting [37, 38]. Physical variables are broadly measurable at the topographic and even micro scales at which many species interact with the environment. Hence, they are important for understanding how species’ ecological niches translate geographically into fine-scale distributions within the physiographic setting. Such resolution issues pertaining to scale have an immense effect on identifying climate change refugia and opportunities for maintaining connectivity [18]. In addition, the influence of physiographic setting on the fine-scale distributions of plant species further determines habitat composition and structure for many animal species. Combined, the fine-scale patterning and partitioning of species distributions within a physiographic setting affect overall richness and the likelihood of stochastic extirpation through the area-heterogeneity tradeoff [39].

Our work to characterize environmental heterogeneity was guided by three criteria. First, we excluded factors that have been used to define physiographic units [7,28,29,40] that change relatively rapidly, such as climate and biotic interactions. Instead, we used only physical factors that remain relatively stable over centuries to millennia (solar insolation, hillslope position, lithology), recognizing that these physical factors are ultimately shaped by tectonic processes, climate, and biota over longer time scales [41]. The advantages of characterizing landforms and physiographic diversity solely on relatively static, physical variables are that: (a) the ecological interpretation is straightforward and parsimonious because solar insolation, hillslope position, and lithology underlie dynamic processes; (b) it explicitly avoids confounding classification thresholds with contemporary patterns defined on the basis of climatological and biotic distributions, which may change dramatically and in unprecedented ways over a management planning time horizon (10–100 years; [42]); and (c) it provides a basis to evaluate the proportion of current biological and environmental diversity that is explained by stable factors that will not change over a planning time horizon, thus offering a baseline for interpreting the magnitudes and uncertainties of future change.

A second guiding criterion was that estimates of physiographic diversity are sensitive to spatial resolution and extent [43], so we derived landforms using a multi-scale analysis [44]. The multi-scale approach informs a wider range of ecological questions by identifying the scale(s) at which particular physiographic settings are defined and experienced by individual species. Fine-grained features can be locally and regionally important [45] and so the resolution of the data should be as detailed as practically possible to assist managers in making decisions at a local scale, typically identified to be at roughly 10–90 m. Landforms generated from high-resolution datasets are especially important as a way to better “downscale” typical climate-based outputs for ecological applications, which tend to be too coarse to be operational at the local level.

A final guiding criterion was the need to make these data relevant to natural resource managers by generating a parsimonious, easy to explain and interpretable model [46]. Moreover, because conservation in the era of rapid climate and land use change forces us to plan at broader extents [47], we wanted to provide comprehensive data that are consistent across broadly defined regions, such as the U.S. Department of Interior Landscape Conservation Cooperatives (LCCs; [48]).

Our goal in this paper is to describe an ecologically relevant classification and map of landforms and physiographic classes that are suitable for climate adaptation planning. We examined the sensitivity of our landform classes to changes in spatial resolution by comparing datasets generated at 10, 30, 90, 270, and 810 m resolution. We illustrate the utility of our datasets by calculating the amount of explanatory power they have against other common indicators of biodiversity patterns and spatial climatic gradients. We also summarized the patterns of landforms and physiography across the conterminous USA, including by LCCs and conduct a “gap analysis” [49] of level of land protection for landform types. Because there is large uncertainty associated with future climate conditions and even more uncertainty around ecological responses, which likely will include novel communities, we believe providing information about what is likely not to change offers a strong foundation for managers to build robust climate adaptation plans.

## Materials and Methods

We generated three related maps: (1) landforms; (2) physiography (landforms combined with lithology); and (3) physiographic diversity.

### Landforms

We developed a comprehensive classification of landforms based on hillslope position and dominant physical processes. Following work articulating hillslope and soil formation (aka topographic sequences; [50,51]), we distinguished four hillslope positions that form a natural sequence of topographic units along the catena: ridges/peaks (summits), upper slopes (shoulders), lower slopes (foot slopes), and valley bottoms (toe slopes). Next, we differentiated the position within each of these hillslopes as a function of solar orientation to reflect how ecological processes (especially soil moisture and evapotranspiration) are strongly influenced by the intensity of insolation and/or shading [24]. Finally, we identified features at the extremes of hillslope gradients, including areas that are very flat (i.e. areas <2°) or very steep (i.e. “cliffs” >50°).

To quantify hillslope position, we calculated a multi-scale topographic position index (TPI) that measures relative topographic relief [52,53]:

$$TPI=E_{o}-E_{n}$$

where *E*_*0*_ is the elevation in meters at a given location (or cell) from the National Elevation Dataset (originally at 10 m resolution, for the contiguous USA; excluding Alaska, Hawaii, and territories) [54], and *E*_*n*_ is the mean elevation of all cells within a neighborhood specified by radius *r*. Highly positive values are associated with peaks and ridges, while highly negative values are associated with valley bottoms and sinks. Locations with values approaching zero typically occur on uniform hillslopes or on flat lands (e.g., plateaus). We standardized TPI following [55] to better resolve fine-scale features:

$$TPI_{s}=\left(E_{o}-E_{n}\right)/E_{s}$$

where *E*_*s*_ is the standard deviation of elevation for all cells within a neighborhood specified by radius *r*. Previously, TPI has been calculated for broad regions using only a single neighborhood, such as a radius of 200 [6], 500 [56], or 564 m globally [57]. So that our analysis would be less sensitive to a particular scale and following [52], we calculated TPIs at multiple scales, selecting neighborhood sizes that would differentiate relatively small geomorphological features such as local hills or ridges from large mountain peaks and divides, and large, broad valleys across the USA. That is, we calculated TPI using radii *(r)* of 270, 810, and 2430 m using a standard progression [58] in multiples of 3 from the base 10 meter native resolution of the DEM that scales roughly in order of magnitude of area and nest with other data used in this analysis (e.g., derived from Landsat imagery at 30 m). Initially we explored additional larger radii (7.2 and 21.8 km), but found they did not discriminate fine-grain (<1 km) features.

We calculated *mTPIs* as:

$$mTPI_{s}=\left(TPI_{s1}+TPI_{s2}+TPI_{s3}\right)/3$$

The break between the upper slope and mid-slope is found where *mTPIs* = 0. Hills were differentiated as upper slopes that had between 30–300 m relief (measured as the elevation gain from its base to highest point; [59]). Ridges/peaks were at least 300 m, and mountains or divides (ridges or peaks that have regional significance) also had at least 300 m relief, but for TPIs where *r* = 2430 m.

Valley bottoms were distinguished from lower slopes where *mTPIs* < -0.75, adjusted from -1.0 to reflect the asymmetrical distribution of elevation values (due to gravity). Narrow and relatively deep valleys (canyons) were found where *mTPIs* < -1.2 and the absolute relief exceeded 5 meters [60]. We also used slope to distinguish two special classes: cliffs were identified as those locations with greater than 50° slope, and flats were identified as areas that have slopes less than 2° when calculated ~810 m resolution to filters out small “speckles” and stripes that can occur from artifacts in digital elevation models. We confirmed these thresholds by visually comparing cliff and flat landforms to 30 m datasets on hillshade and delineated geomorphological types from a high resolution soils database (http://tinyurl.com/ssurgo), at 3 random locations in nearly 60 ecoregions across the USA.

We used an estimate of incident radiation and heat load [61] that combines slope, aspect, and latitude to predict the ecological effects of potential direct radiation. In contrast to other methods that have relied on aspect [7,36] or slope and solar insolation [52,62], heat load has a strong empirical relationship to evapotranspiration that controls vegetation distributions [56,61] and can be readily calculated at broad extents and high resolution (30–90 m).

We modified the original heat-load index [61] in two ways to construct a continuous heat-insolation load index (CHILI). First, we modified the formula to explicitly incorporate latitude as a variable so that heat load is calculated on a continuous (latitudinal) basis, assuming insolation at the equinox [52]. Second, the original heat-load index is based on a 45° “folding” to mimic patterns of evapotranspiration and measured heat load that increase in the afternoon. We modified the folding to 22.5°, based on more recent empirical studies that found that thermal south ranged from ~10° to 30° west of south over the growing season [63], and mean maximum daily temperatures were reached at ~20°-45° west of south [64]. We did not include assumptions that might influence the amount of radiation due to atmospheric conditions (e.g., due to clouds) or other specific assumptions about growing seasons.

We classified the heat load index to distinguish “warm”, “neutral”, and “cool” portions of a landscape. We established these classes from the CHILI values (warm > 0.767, cool <0.448, neutral 0.448–0.767) by finding the thresholds that resulted in equal areas of each class on a simulated landscape. We did this using an idealized mountain formed by a Gaussian surface, where we controlled the height (i.e., population) to width (i.e., bandwidth) of a kernel density function to minimize areas with either >50° (“cliff”) or <2° (flat). The warm/neutral/cool patterns generated from the thresholds aligned well with independent estimates for a range of physiographic provinces across the USA.

Our final comprehensive classification of landforms consists of 15 classes (Table 1). We implemented the landform algorithm (S1 Fig) using the Google Earth Engine platform [65], at five resolutions (or grains: 10, 30, 90, 270, and 810 m) using a 10 m USGS DEM [54].

**Table 1:** A hierarchical classification of ecologically relevant landforms.

Hillslope position	ID	Class name	TPI	Slope (°)	CHILI
Summit	11	Peak/ridge warm	(0.0 < *mTPIs* < 1.0) and (30 < (*E*_*o*_ *-E*_*n*_) < 300)		Warm
Summit	12	Peak/ridge	(0.0 < *mTPIs* < 1.0) and (30 < (*E*_*o*_ *-E*_*n*_) < 300)		Neutral
Summit	13	Peak/ridge cool	(0.0 < *mTPIs* < 1.0) and (30 < (*E*_*o*_ *-E*_*n*_) < 300)		Cool
Summit	14	Mountain/divide*	(0.0 < *mTPIs* < 1.0) and ((*E*_*o*_ *-E*_*n*_) ≥ 300)		
Summit	15	Cliff		>50	
Upper slope	21	Upper slope warm	(0.0 < *mTPIs* < 1.0) and ((*E*_*o*_ *-E*_*n*_) ≤ 30)		Warm
Upper slope	22	Upper slope neutral	(0.0 < *mTPIs* < 1.0) and ((*E*_*o*_ *-E*_*n*_) ≤ 30)		Neutral
Upper slope	23	Upper slope cool	(0.0 < *mTPIs* < 1.0) and ((*E*_*o*_ *-E*_*n*_) ≤ 30)		Cool
Upper slope	24	Upper slope flat	(0.0 < *mTPIs* < 1.0) and ((*E*_*o*_ *-E*_*n*_) ≤ 30)	<2	
Lower slope	31	Lower slope warm	(-0.75 < *mTPIs* < 0.0) and ((*E*_*o*_ *-E*_*n*_) > -5)		Warm
Lower slope	32	Lower slope neutral	(-0.75 < *mTPIs* < 0.0) and ((*E*_*o*_ *-E*_*n*_) > -5)		Neutral
Lower slope	33	Lower slope cool	(-0.75 < *mTPIs* < 0.0) and ((*E*_*o*_ *-E*_*n*_) > -5)		Cool
Lower slope	34	Lower slope flat	(-0.75 < *mTPIs* < 0.0) and ((*E*_*o*_ *-E*_*n*_) > -5)	<2	
Valley bottom	41	Valley	(*mTPIs* < -0.75)		
Valley bottom	42	Valley (narrow)	(*mTPIs* < -1.2) and ((*E*_*o*_ *-E*_*n*_) ≤ -5)		

Classes were based on dominant hillslope position and defined using the topographic position index (TPI), slope, and continuous heat load index (CHILI).

*Difference in elevation calculated at r = 2430, others are calculated at r = 810. ID is the unique identifier used to label each class in the landform dataset.

### Lithology

We used the best-available national-level dataset on lithology [66], which specifies 18 ecologically relevant parent material classes defined by textural and chemical similarities and mapped as polygons consistently across the USA (1:1,000,000). Rather than soil class information, we used geologic parent material because of the strong linkage between these characteristics and ecological response [7]. That is, in addition to differentiating crude soil texture categories, lithology incorporates differences in chemical properties found to be important to vegetation. We converted the polygons to a 270 m resolution raster to differentiate linear, narrow (~540 m in width) polygons. We then overlayed the lithology classes with the land form classes, generating the physiography dataset at 270 m resolution. We explored the higher spatial resolution SSURGO (http://tinyurl.com/ssurgo) and STATSGO (http://www.tinyurl.com/statsgo) soils databases, but these had numerous inconsistencies in classifications, were incomplete in roughly 20% of the USA (with higher proportions of missing classes in terrain with higher relief), and had attributes on parent material available for only roughly 10% of the USA.

### Multi-scale diversity

We estimated environmental heterogeneity by calculating the diversity of physiographic units using Shannon’s equitability (*E*_*H*_; 0 to 1), which is calculated by normalizing the Shannon-Weaver diversity index (*H*):

$$E_{H}=\frac{H}{H_{\text{max}}}=H/\text{ln}(S)$$

$$H=-\sum_{i=1}^{s}\left(p_{i}\text{ln}p_{i}\right)$$

where *p*_*i*_ is the proportion of observations (cells) of type *i* in a given neighborhood and *S* is the number of physiographic types. We used a multi-scale approach to calculate the diversity of types using moving-window neighborhoods based on the average area of hydrologic unit codes 4, 6, 8, 10 12, 14, and 16 (i.e. 115.8, 89.9, 35.5, 13.1, 5.6, 2.8, and 1.2 km radius). We used the area of hydrologic units as the basis for the multi-scale moving window analysis because they are hierarchically organized and represent ecologically relevant scales [67]. Note that the resolution (or grain) of the diversity dataset is 270 m based on the physiography data layer described above. We conducted these spatial analyses using ArcGIS v10 (Esri 2014, Redlands, CA).

### Relationship with existing indicators

To examine some of the linkages between the physiographic setting and climate adaptation strategies, in accordance with our starting criteria, we asked a series of questions that could be answered using a combination of our landform and physiographic diversity datasets, as well as others commonly used to understand patterns of biodiversity and climatic variation. Our first question related strongly to the strategy to *protect key ecosystem features*: What proportion of variation in biodiversity is explained by variation in physiographic setting? To assess the relationship with patterns of biodiversity, we examined physiographic diversity in relation to vertebrate species richness [68] and ecological system richness [69]. Range maps for USA mammals (91 species) and amphibians (n = 48) and bird breeding ranges (n = 200) were compiled at 10 km resolution. We calculated richness as the multi-scale average of the number of species using the same watershed-based scales described earlier. The multi-scale average of the number of ecological systems (579 vegetation types) was calculated from 30 m resolution data [67].

Because the environmental heterogeneity that influences key ecosystem features is influenced by a combination of climatic and physiographic factors, we posed a second related question: What proportion of variation in current meso-scale climate (840 m resolution) was explained by variation in physiographic setting? We answered this question in two ways. First, to assess the relationship between our physiographic datasets and meso-climatic variation, we compared landform diversity against historical temperature variability. Landform diversity was calculated as the average of the landform *E*_*H*_ (at 840 m resolution) across moving windows for the multi-scale watersheds. Temperature variability was calculated as the multi-scale average of the standard deviation in historical (1950–2000) annual mean temperature from WorldClim [70], also at 840 m resolution. Second, we compared spatial variation between *mTPI* and historical estimates of meso-climate from WorldClim, where slope (rise/run) was calculated using the average maximum technique [22]. We calculated slopes on the raw *mTPI* values (before classifying into slope position categories; m/km) and the denominator of climate velocity (°C/km).

In answering the above questions, we used Pearson’s product-moment correlation coefficients [71] to assess how much variation in the indicator response variables was explained by the landform and physiographic predictor variables. Our interest was to simply quantify the proportion of current environmental heterogeneity that is explained by stable factors that change relatively little over a planning time horizon.

## Results

We first analyzed how our landform classes were sensitive to resolution, and found that general trends in class proportions were consistent throughout the USA over a range of scales (resolution), but strong differences for classes that occupied small portions of the USA (Table 2) were evident as well. We found a more than 2-fold difference for: hill/ridge (cool) and cliff (30:90 m ratio); upper slope (cool) and lower slope (cool) (30:270 m ratio); and upper slope (warm), upper slope, and lower slope (warm). Interestingly, we found that “cool” landforms that are likely to be least affected by warming climates [19] were particularly sensitive to resolution and are under-represented at coarser-resolutions. The valley bottom (narrow) class showed directional changes, first slightly decreasing in representation as a function of coarser resolution, but at 810 m exhibited a 25% increase in representation (compared to 30 m).

**Table 2:** Landform classes in the conterminous USA at different resolutions.

	Percent in each class at resolution (m)				Ratio		
Name	30	90	270	810	30:90	30:270	30:810
Hill/ridge (warm)	0.31%	0.31%	0.38%	0.48%	1.00	-1.24	-1.57
Hill/ridge	0.69%	0.73%	0.66%	0.64%	0.94	1.03	1.07
Hill/ridge (cool)	0.05%	0.01%	0.00%	0.00%	4.03	51.51	6318.84
Peak/divide	0.05%	0.05%	0.05%	0.07%	-1.01	-1.02	-1.51
Cliff	0.07%	0.02%	0.01%	0.00%	3.18	11.68	n/a
Upper slope (warm)	12.40%	11.39%	9.34%	5.04%	1.09	1.33	2.46
Upper slope	14.32%	13.05%	11.30%	7.05%	1.10	1.27	2.03
Upper slope (cool)	0.69%	0.39%	0.13%	0.01%	1.77	5.48	96.50
Upper slope (flat)	18.19%	19.61%	23.41%	33.50%	-1.08	-1.29	-1.84
Lower slope (warm)	12.23%	11.04%	9.30%	5.80%	1.11	1.31	2.11
Lower slope	10.99%	10.29%	9.96%	6.84%	1.07	1.10	1.61
Lower slope (cool)	0.41%	0.23%	0.09%	0.00%	1.80	4.70	83.54
Lower slope (flat)	20.16%	23.45%	25.80%	28.75%	-1.16	-1.28	-1.43
Valley bottom	8.29%	8.30%	8.48%	10.41%	1.00	-1.02	-1.25
Valley bott. (narrow)	1.14%	1.13%	1.09%	1.43%	1.01	1.05	-1.25

This table shows the proportion of the conterminous USA by landform class, as a function of resolution.

**Fig 1:**
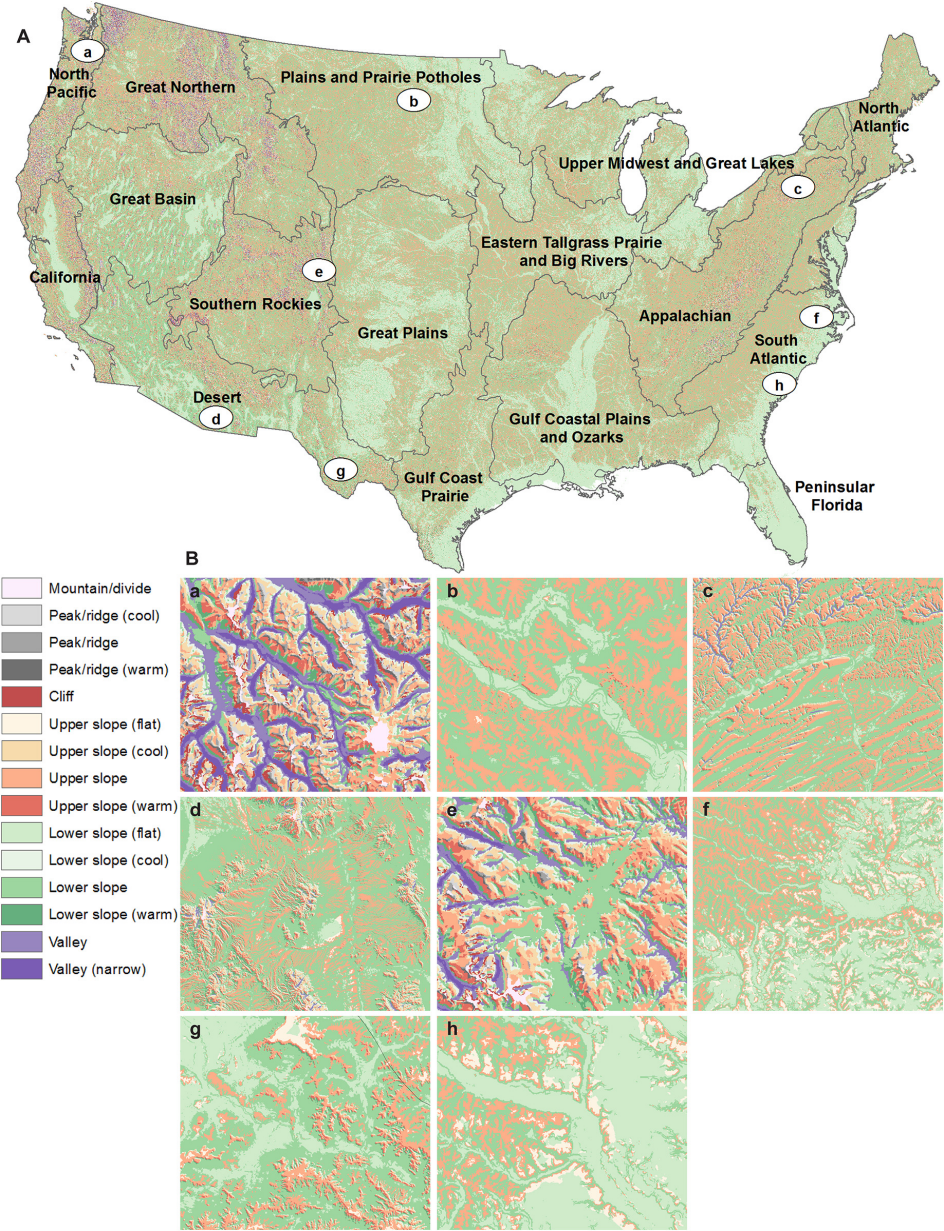
Landforms of the conterminous USA. (A) A landform map of the USA, with Landscape Conservation Cooperatives used by the Department of Interior to guide climate change adaptation. Labels a-h refer to inset examples and legend for class types. (B) Examples of landform classes, zoomed in to illustrate different patterns: (a) the Pacific Northwest around Mount St. Helens (1:175,000); (b) along the Missouri River at the boundary of Montana and North Dakota (1:200,000); (c) near Milton, Pennsylvania (1:500,000); (d) in the Sky Islands of southern Arizona (1:500,000); (e) Estes Park, Colorado (1:175,000); (f) near Smithfield, North Carolina (1:400,000); (g) along the Ogeechee River near Statesboro, Georgia (1:300,000); and (h) south Texas tablelands (1:200,000).

Because we found that landforms were clearly sensitive to spatial resolution, we provide our results below based on the 30 m resolution dataset. Some general patterns are evident by examining the variation in the proportion of each class within each LCC across the USA (Fig 1A; Table 3), but landforms also exhibit complex local patterns that affect climate heterogeneity (Fig 1B). By hillslope position, we found that 1.16% of the conterminous USA is considered to be peaks/divides and hills/ridges, 45.61% on upper slopes, 43.79% on lower slopes, and 9.44% in valley bottoms. “Neutral” heat load dominates most hillslope position classes. As a proportion of the USA, hills/ridges had 0.69% in neutral heat settings, while 0.31% was warm and only 0.05% is cool. Upper slopes were dominated as well by “neutral” heat load situations (with 14% neutral and 18% flat, with 12% “warm” and 0.7% “cool”). Lower slopes also were dominated by neutral heat loads with 11% neutral and 20% flat, with 12% warm and 0.4% cool. Finally, deep and narrow valleys cover 1.14%, while broad valley bottoms cover 8.29% of the USA.

**Table 3:** Ecoregional distribution of landforms.

	Area	Summit					Upper slope				Lower slope				Valley bottom	
LCC	(km^2^)	Peak/ridge warm	Peak/ridge neutral	Peak/ridge cool	Mtn/divide	Cliff	Warm	Neutral	Cool	Flat	Warm	Neutral	Cool	Flat	Valley	Narrow
North Pacific	179,408	0.369	2.885	0.100	0.278	0.027	10.02	27.98	2.79	2.80	11.20	24.61	1.61	5.77	8.33	1.23
California	210,173	1.046	1.384	0.007	0.125	0.039	18.21	13.32	0.34	9.21	21.17	10.00	0.22	16.81	7.38	0.74
Great Northern	660,379	0.124	1.791	0.098	0.245	0.087	6.48	30.04	2.92	4.89	7.43	27.12	1.85	8.83	7.18	0.92
Great Basin	562,700	0.319	1.133	0.009	0.070	0.010	8.49	17.12	0.35	9.90	13.34	17.68	0.15	26.63	4.43	0.36
Gulf Coast Prairie	382,730	0.141	0.015	0.000	0.000	0.000	12.10	0.11	0.00	34.28	9.28	0.04	0.00	34.07	9.20	0.76
Southern Rockies	516,755	0.645	1.136	0.016	0.103	0.148	21.34	14.85	0.47	7.29	22.17	11.75	0.23	12.51	6.48	0.86
Plains and Prairie Potholes	783,896	0.008	0.265	0.002	0.001	0.000	0.44	20.34	0.02	25.21	0.57	15.93	0.01	27.38	9.00	0.82
Great Plains	782,004	0.041	0.054	0.000	0.000	0.000	7.44	6.12	0.00	34.42	6.44	3.87	0.00	32.90	7.77	0.93
Upper Midwest & Great Lakes	540,064	0.011	0.226	0.000	0.001	0.000	0.88	15.70	0.00	28.55	0.90	10.22	0.00	32.85	9.44	1.20
Eastern T. Prairie & Big Rivers	535,630	0.010	0.026	0.000	0.000	0.000	6.30	11.15	0.00	31.46	5.41	6.06	0.00	28.30	9.84	1.45
Gulf Coast Prairie	382,730	0.141	0.015	0.000	0.000	0.000	12.10	0.11	0.00	34.28	9.28	0.04	0.00	34.07	9.20	0.76
Gulf Coastal Plains and Ozarks	728,639	0.234	0.150	0.000	0.000	0.000	20.66	2.12	0.00	22.17	14.30	1.07	0.00	26.98	10.71	1.61
Appalachian	592,906	1.007	2.061	0.001	0.007	0.000	22.80	17.66	0.06	4.36	20.20	11.45	0.02	6.81	11.08	2.50
North Atlantic	289,740	0.146	1.127	0.006	0.009	0.000	8.01	23.51	0.10	13.02	6.96	20.53	0.02	16.52	8.80	1.24
South Atlantic	357,847	0.060	0.041	0.000	0.000	0.000	18.96	0.45	0.00	29.80	13.38	0.20	0.00	24.91	10.08	2.12
Peninsular Florida	93,383	0.002	0.000	0.000	0.001	0.000	0.73	0.00	0.00	43.81	0.36	0.00	0.00	44.48	9.59	1.04

This table shows the percentage of each landform class in the conterminous USA by Landscape Conservation Cooperative ecoregions (LCC).

**Fig 2:**
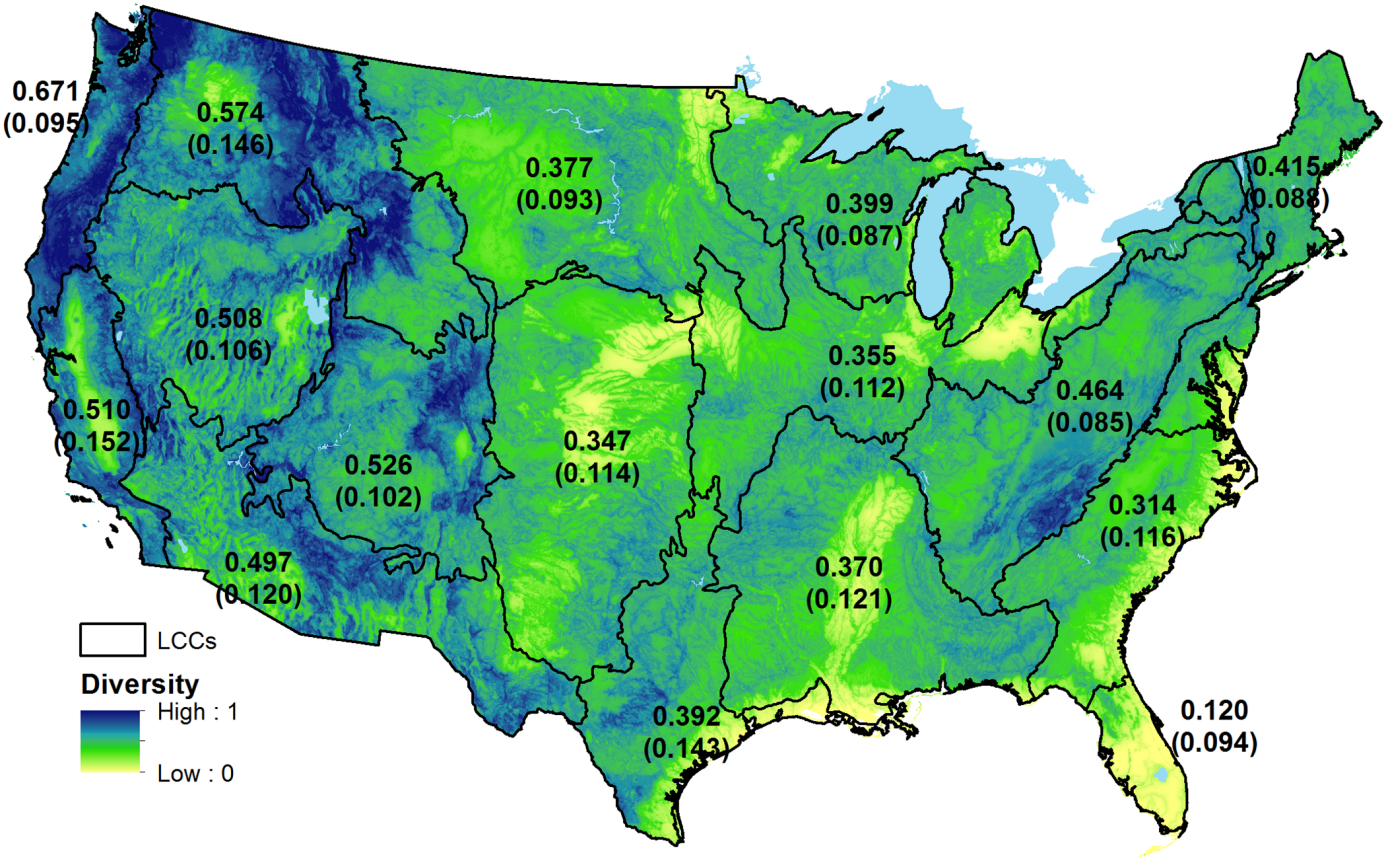
Physiographic diversity in the conterminous USA. Multi-scale physiographic diversity, calculated using the Shannon-Weaver index. Labels are mean diversity by Landscape Conservation Cooperatives, with standard deviations in parentheses.

We overlaid the 15 landform types with 18 classes of soil parent material, resulting in 269 unique combinations of physiographic types (270 possible; Fig 2; Table 4). The multi-scale physiographic *E*_*H*_ averaged 0.382 (SD = 0.068), with high mean values located in the North Pacific (0.671), Great Northern (0.574), and Southern Rockies (0.526), and low mean values located in Peninsular Florida (0.120), South Atlantic (0.314), and the Gulf Coast Prairie (0.321), and the Great Plains (0.347). Strong patterns were evident and coincide with major mountain ranges of the Cascades, Bitterroot/Absaroka, Sierra Nevada, Southern Rockies, and the Appalachians. High diversity also occured in areas with major river systems, throughout the Midwest in particular.

**Table 4:** Parent soil material types by landform class in the conterminous USA.

	Summit	Peak/ridge				Upper slope				Lower slope				Valley bottom		
Parent material	Mtn/divide	Cool	Neutral	Warm	Cliff	Flat	Cool	Neutral	Warm	Flat	Cool	Neutral	Warm	Valley	Narrow	%
Water areas	0	116	2	2	0	2,756	2,446	39	6,663	4,738	4,652	70	44,408	5,819	300	**2.84%**
Carbonate	3,041	3,821	69	427	436	107,376	39,875	2,397	56,633	87,812	26,630	1,313	53,079	38,899	6,949	**5.38%**
Non-carbonate	6,470	17,207	413	1,103	689	301,360	310,013	10,669	266,030	266,910	250,832	5,696	301,094	158,182	21,688	**24.12%**
Alkaline intrusive	30	86	0	6	1	1,285	3,635	23	865	1,280	2,753	14	1,093	645	140	**0.15%**
Silicic residual	7,051	16,749	460	1,981	531	175,353	194,703	15,058	28,853	175,545	147,859	8,997	26,370	60,104	10,510	**10.94%**
Extrusive volcanic	212	552	12	32	10	7,400	13,656	289	17,639	7,274	11,073	153	22,492	3,433	289	**1.06%**
Colluvial sediment	5,098	9,865	5	38	4	130,028	102,240	283	56,663	112,038	62,194	109	54,174	63,951	13,855	**7.68%**
Glacial till—clay	3	29	0	0	0	411	5,053	0	42,074	437	3,339	0	38,469	8,079	784	**1.24%**
Glacial till loamy	251	2,090	6	7	1	26,116	133,142	171	293,759	23,075	84,497	91	229,810	79,783	10,866	**11.12%**
Glacial till coarse	356	3,183	32	39	8	9,742	56,763	656	6,289	12,025	53,989	459	13,285	14,451	1,087	**2.17%**
Glacial lake sediment fine textured	28	457	11	7	4	2,402	31,878	542	79,935	3,476	29,812	553	107,186	29,783	3,500	**3.65%**
Glacial outwash coarse textured	20	184	2	1	1	1,550	12,353	40	40,864	1,713	10,909	57	56,492	13,173	1,130	**1.76%**
Hydric, peat, muck	0	2	0	0	0	52	321	0	19,683	27	241	0	24,215	4,903	215	**0.63%**
Eolian sediment (coarse)	161	266	1	2	10	12,200	19,100	13	114,581	11,286	12,020	4	113,351	21,676	2,146	**3.86%**
Eolian sediment (fine)	24	209	1	0	1	14,876	40,851	63	74,135	11,921	25,201	28	56,507	20,675	3,112	**3.11%**
Saline lake sediment	74	288	2	10	2	3,010	4,744	62	17,498	7,576	9,444	28	65,828	5,329	357	**1.44%**
Coastal fine-textured sediment	1,136	1,689	12	17	18	92,631	48,071	338	377,499	134,719	67,369	410	598,242	113,265	10,804	**18.08%**
Coastal coarse-textured sediment	1	6	0	0	0	369	63	0	31,266	201	59	0	24,049	5,420	733	**0.79%**
**%**	**0.31%**	**0.73%**	**0.01%**	**0.05%**	**0.02%**	**11.39%**	**13.05%**	**0.39%**	**19.61%**	**11.04%**	**10.29%**	**0.23%**	**23.45%**	**8.30%**	**1.13%**	**100.00%**

This table shows the area (km^2^) in the conterminous USA for each type of parent soil material type by landform class.

**Fig 3:**
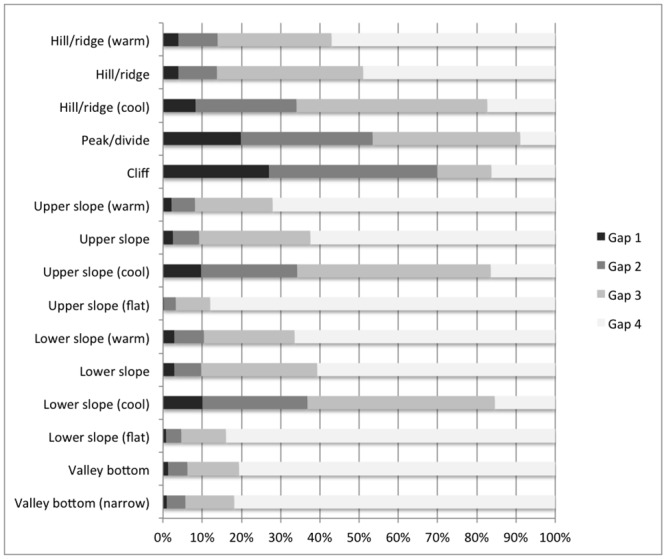
Level of land protection by landform class. Results from a “gap” analysis of landforms showing the level of land protection for each land form class. Level of protection follows the gap status classes [48]: Status 1—permanent protection from conversion and natural disturbances; Status 2—permanent protection from conversion but may have some modification of natural disturbances; Status 3—protection from conversion for most areas but extractive uses may be allowed; and Status 4—no known formal legal mandates or restrictions to prevent conversion of natural habitat types.

We also examined landform classes in relation to protected areas (aka a “gap analysis” [48]; Fig 3). Not surprisingly, we found that the highest levels of protection tended to occur on peaks/divides and hills/ridges, while the lowest level of protection occurred at lower slope and valley bottoms locations. Only 5 of 15 landform types were represented at the Aichi Biodiversity Target level (17%): peak/divide, cliff, hill/ridge (cool), upper slope (cool), and lower slope (cool).

We found varying levels of explanatory power among our variables and other indicators of biodiversity commonly used in climate adaptation planning. Overall, the Pearson’s product-moment correlation between physiographic diversity and vertebrate species richness was 0.449, while the correlation with ecological systems was 0.285. The strongest correlation we found was between slopes of topographic position and the denominator of climate velocity (0.792). The correlation between landform diversity (excluding parent material) and temperature variability (SD) was 0.483.

## Discussion

The conterminous USA exhibits a wide range of landforms and parent material, resulting in considerable physiographic diversity. Our maps of landform classes and physiographic diversity are novel contributions that characterize ecologically relevant aspects of abiotic environmental heterogeneity. These databases provide a consistent and comprehensive platform for climate adaptation planning at local to regional and national scales.

Climate adaptation planning will likely involve pursuing multiple strategies, and below we briefly explore how landforms and physiographic diversity could inform such efforts—especially *protecting key ecosystem features*, *ensuring connectivity*, *supporting evolutionary potential*, and *protecting refugia*. Note that our intent is to provide a few illustrative examples of how our datasets can be applied within an adaptation framework, not to argue that these are the only datasets or uses. Indeed, we expect future research will further explore the use of physiographic variables for climate adaptation planning, especially to evaluate indicators and available datasets, and to compare and evaluate data gaps.

We found that our measures of physiographic diversity had moderate levels of explanatory power related to current patterns of biodiversity (0.449 for species richness), suggesting that our physiographic datasets meaningfully capture *key ecosystem features* that structure biodiversity and can aid coarse-filter approaches [5] to conservation planning. Our datasets may also be extended to support “fine-filter” conservation planning and vulnerability assessments focused on a particular ecosystem feature as a conservation target (e.g., an individual species or important habitat). Fine-filter approaches might instead select particular spatial extents relevant to the conservation target or particular landform or physiographic classes with which a given species has high affinity. These may be used to better understand the fine scale distribution of particular species, as well as the topographic and micro environmental influences of climate and other important environmental variables influencing a species niche. Similarly, our physiographic dataset can be leveraged to help *ensure connectivity* for fine-filter conservation targets by identifying potential movement pathways among physiographic classes or to identify locations of key landform types that facilitate ecological flows, such as slopes and valley bottoms [72]. Likewise, it can be used to identify locations for *protecting refugia*, which might be located on cool slopes or valley bottoms where cold-air pooling has potential to occur [19].

We found that the spatial rate of change in *mTPI* (m/km) was highly correlated with the spatial rate of change in temperature (°C/km) (0.792), suggesting that our physiographic diversity dataset is predictive of areas with strong temperature gradients. Importantly, we expect the relationship to be scale dependent [73], but also note that our *mTPI* was calculated over a range of biologically relevant scales (270 to 2430 m) that encompassed meso-climate (840 m). The presence and location of strong climatic and edaphic gradients identified by our physiographic diversity dataset would provide information useful for strategies aiming to *support evolutionary potential*, given the importance of these gradients to evolutionary processes such as diversification, speciation, and extinction [74–76].

An important application of landforms is to “place” coarser-scale climate change information to allow resource managers to understand how a particular species or ecological process might respond to climate change at a finer scale. There is a strong theoretical basis for understanding the relationship of landform types to climate [19]. Although geologic parent material has a less clear role, it can indirectly influence climate through soil characteristics (e.g., productivity and water-holding capacity) and—over shorter time scales—vegetation [77].

Not surprisingly, we found a strong scaling effect on the amount and especially pattern of landform classes as a function of resolution (Table 1. The interaction between physiography and climate is highly scale dependent [18]–and it is important to note that most climate information is still relatively coarse (~1 km is considered highly “downscaled” climate data). Our findings that landforms that are most likely to be related to climate refugia (“cool” and valley bottom) are especially sensitive to scaling suggests that work on identifying these areas should consider representation and patterning effects of scale. Recent work on micro-scale climatology has found that environmental heterogeneity varies in ecologically significant ways at horizontal scales as small as 1 m^2^, and vertical scales even smaller at 1 cm^2^ [78,79]. Currently these resolutions are too small to be mapped in a national-level database, but may be feasible for localized studies. More generally, opportunities exist to develop stronger linkages between geomorphology and vegetation by building in explicit ecological mechanisms and processes, including soils and other climate measures that may reflect physiological tolerances (e.g., maximum daily temperature), but also insolation (slope and aspect) and resulting water-balance [78]. Because the lithology dataset is a relatively coarse scale (~1:1,000,000; converted to 270 m resolution), we recommend limiting interpretation of physiographic patterns to features at least 1 km^2^ in size..

Many agencies and organizations are engaging in large landscape conservation and general guidance has begun to emerge on how to conduct and coordinate climate adaptation planning [2,11,30,80–82]. Although analyses of climate impacts on conservation targets are a key component of most climate adaption planning frameworks, there is little guidance on how to address broad, landscape-level impacts [18,80]. Rather, in contrast to species or habitat-level conservation, the physiographic approach provides insight into the enduring features that will continue to structure biodiversity in the face of climate change. We recognize that different regional conservation groups, such as the LCCs, will likely need to adopt different strategies for adapting to climate change, to allow for regional differences in climate change impacts, but they may consider our landform and physiographic diversity datasets as a foundation for pursuing climate adaptation strategies on where and how to invest in climate adaptation.

## Supporting Information

S1 Fig Data flow diagram of landforms. Landforms were defined using basic topographic measures derived directly from the USGS 10 meter Digital Elevation Model, as well as latitude. Physiographic classes were generated by overlaying the landforms and lithology (parent material) converted from polygons (1:1,000,000 scale) to 270 m raster grid. The program used to generate these datasets is available from the authors upon request.
(PNG)
