# Supplementary figures and images for: Ecologically-Relevant Maps of Landforms and Physiographic Diversity for Climate Adaptation Planning
Source: PLoS One. 2015 Dec 7;10(12):e0143619. doi: 10.1371/journal.pone.0143619 (PMC4671541; doi:10.1371/journal.pone.0143619)

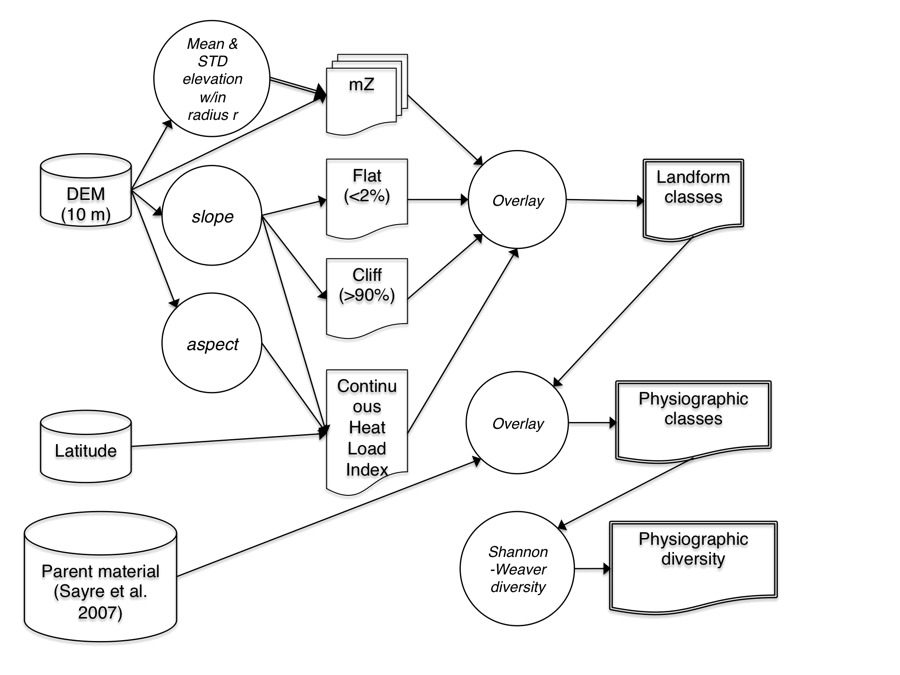

Supplement: S1 Fig — Landforms were defined using basic topographic measures derived directly from the USGS 10 meter Digital Elevation Model, as well as latitude. Physiographic classes were generated by overlaying the landforms and lithology (parent material) converted from polygons (1:1,000,000 scale) to 270 m raster grid. The program used to generate these datasets is available from the authors upon request. (PNG) [file pone.0143619.s001.png]
